# T Cell Microvilli: Sensors or Senders?

**DOI:** 10.3389/fimmu.2019.01753

**Published:** 2019-07-30

**Authors:** Hye-Ran Kim, Chang-Duk Jun

**Affiliations:** ^1^School of Life Sciences, Gwangju Institute of Science and Technology, Gwangju, South Korea; ^2^Immune Synapse and Cell Therapy Research Center, Gwangju Institute of Science and Technology, Gwangju, South Korea

**Keywords:** microvilli, T cell microvilli particle, dendritic cells, cell communication, trogocytosis

## Abstract

Communication between cells is essential for multicellular life. During cognate immune interactions, T cells communicate with antigen-presenting cells (APC) via direct cell–cell contact or the release of molecules and vesicles containing T cell messages. A wide variety of mechanisms have been reported and among them a process called “trogocytosis” has traditionally been thought to be the fastest way to directly transfer membrane portions containing intact proteins from one cell to another; however, the mechanism is unverified. Trogocytosis has been distinguished from the generation of extracellular vesicles (EVs), a term that encompasses exosomes and microvesicles, as EVs are released via a contact-independent manner and are suggested to potentially send molecular messages over a distance. However, some previous reports regarding EVs in T cells may be misleading in terms of explaining their cellular origins. In addition, there is little evidence on how EVs are generated from T cells *in vivo* and function to regulate complex immune responses. A recent work demonstrated that T cell microvilli—thin and finger-like membrane protrusions—are highly fragile and easily separated as membrane particles by trogocytosis, forming a new class of EVs. Surprisingly, released T cell microvilli-derived particles act as vectors, transmitting T cell messages to cognate APCs. This review focuses on how T cell microvilli vesicles are connected with immune regulation mechanisms discovered previously.

## Highlights

- T cell microvilli have been supposed to act as sensory organs to survey surfaces of APCs.
- T cell microvilli are highly fragile to be separated in a process of trogocytosis.
- Separated large T-cell microvilli particles (TMPs) contain TCRs and membrane budding complexes.
- Large TMPs are further fragmented to the exosome-sized TMPs, forming new class of extracellular vesicles.
- T cell microvilli constitute “immunological synaptosomes” that act as T cell messengers.

## Introduction

Information exchange between T cells and cognate antigen-presenting cells (APCs) dictates the character and scope of immune responses. Thus, understanding the mechanisms of the message transfer between two cells has been a subject of constant interest. Among the various modes of message transfer, the immunological synapse (IS) is the most aggressively studied architecture of communication formed between T cells and APCs, which was discovered approximately two decades ago (1, 2). Within an IS, many molecules involved in T cell activation or deactivation that compose typical clustering patterns called supramolecular activation clusters (SMACs) can be exchanged. Some scientists argue that ISs provide a platform for TCR sorting and release TCR-enriched microvesicles to the cognate APCs (3). However, the mechanism by which T cells release TCR-enriched microvesicles still remains controversial based on the recent works described at the last paragraph of this section (4, 5).

Over two decades before the IS was discovered, scientists made surprising *in vitro* and *in vivo* observations; proteins thought to be specific for one cell type were found in small amounts on the surfaces of other cell types (6–8). This process has been referred as “absorption” (9), “internalization” (10), or “trogocytosis” (11, 12) (from the ancient Greek “trogo,” meaning “gnaw” or “nibble”) and has characteristics distinct from enzyme-mediated cleavage or exosomal transfer (12, 13). Trogocytosis has traditionally been thought to be the fastest way to directly transfer membrane portions containing intact molecules from one cell to another. However, the functional consequence and the mechanism of trogocytosis have not been clearly verified, while many studies have collectively indicated that the process can have a potential role on the course of immune responses (12, 13). Membrane nanotubes—long membrane tethers between cells—are also readily observed and can connect a wide variety of cells, including T cells, B cells, and innate immune cells such as NK cells and macrophages (14–18). Nanotubes can allow the intercellular exchange of molecules as well as signals; however, there is no definitive evidence of nanotube-mediated molecular exchanges between immune cells. Recently, extracellular vesicles (EVs) have attracted attention as they contain proteins as well as genetic materials such as small RNA, and have been implicated in immune responses related to tumors, allergies, and autoimmune diseases (12, 13, 19–21). However, although the generation processes of EVs are fairly well identified *in vitro*, their origins *in vivo* have yet to be clarified due to the current limitations of the technology used to trace EVs. In addition, the molecular compositions of EVs are heterogeneous, and thus there are no ideal ways to accurately distinguish the origins of EVs in each experiment. Indeed, size exclusion is not the standard method for classifying the origin of EVs (22). Overall, currently suggested mechanisms of molecular transfer between T cells and APCs are vague, and some mechanisms may be used interchangeably.

Recently, our group and Cai et al. identified that T cell microvilli are highly dynamic and polarized onto the surface of antigen-bearing APCs, suggesting their roles in scanning and sensing the antigens on APCs (5, 23). In line with this, a recent super-resolution microscopy study demonstrated that TCRs are highly condensed in microvilli tips, emphasizing that these surface projections are effective sensors for antigenic moieties on APCs or target cells (4). Strikingly, we immediately found that microvilli are separated from the T cell body by the combined action of two independent mechanisms, trogocytosis and membrane budding, and are deposited on the surface of cognate antigen-bearing APCs, thus potentially acting as the most efficient and effective means to deliver T cell messages to cognate APCs. The final size of T cell microvilli particles (TMPs) is comparable to that of exosomes (40–100 nm) (5). The current evidences in my laboratory suggest that some previous studies may need to be revisited to clarify whether any phenomenon was misinterpreted or if the same phenomenon was interpreted from different perspectives. Here, we focus on the T cell microvilli and their roles in molecular and cellular aspects, especially in relation to the IS and TCR clusters, trogocytosis, membrane nanotubes, and EVs.

### Lymphocyte Microvilli

**Figure 1:**
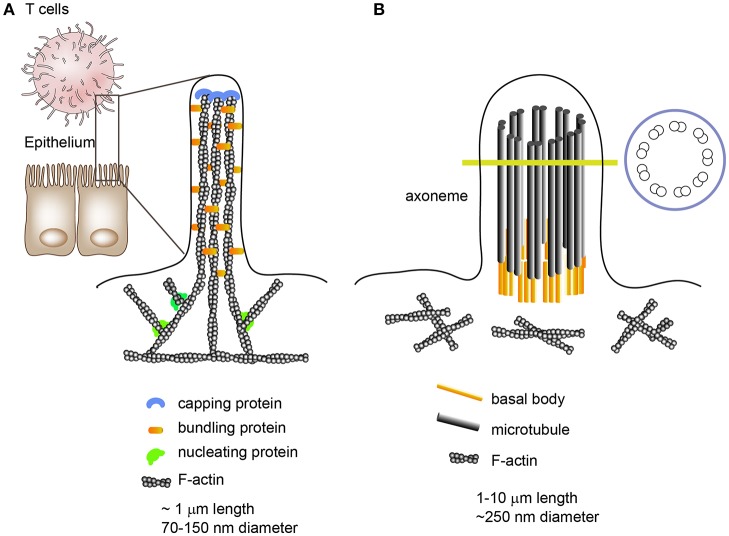
Morphologies and internal structures of microvilli, filopodia, and cilia/flagella. **(A)** Microvilli are cellular membrane extensions which consist of cytoplasm and cross-linked actin filaments. Epithelial cell microvilli are specialized for nutrient absorption; however, lymphocyte microvilli are more like filopodia, which extend and shrink intermittently via the alternate assembly and disassembly of their actin filaments. Filopodia are slender cytoplasmic projections which contain actin filaments cross-linked into bundles by actin cross-linking proteins. **(B)** Unlike microvilli or filopodia, cilia contain a microtubule-based cytoskeleton known as the axoneme.

Microvilli are outer membrane organelles that vary between 0.1 μm and several micrometers in length and 70–150 nm in diameter (24). Microvilli contain cytoplasm and microfilaments; however, cellular organelles are nearly absent in microvilli. Structurally, each microvillus contains cross-linked filamentous actin bundles that are laterally connected by several bundling proteins, such as fimbrin (or plastin-1) or villin (Figure 1). In general, microvilli on intestinal epithelial cells maintain a constant length and are specialized for cell-surface enlargement, which facilitates nutrient absorption (25). In contrast, lymphocyte microvilli have characteristics similar to those of filopodia, which grow and shrink intermittently via the alternate assembly and disassembly of their actin filaments (Figure 1) (26). Moreover, the number and length of T cell microvilli are dependent on the state of the T cells; for instance, the diameter of effector T cells is approximately twice as large as resting T cells and they have longer microvilli, although their mean diameter is similar to that of resting T cells (4, 27).

The general importance of microvilli has been strongly underestimated and the essential functions of these finger-like membrane protrusions in T cells remain largely unrecognized. The structural and biophysical features of these abundant surface organelles imply important functional competences. For instance, microvilli tips provide a platform for the segregation of many functionally important membrane proteins, enabling the clustering of large organized complexes of signaling molecules that allow highly effective, rapid, and spatially defined signal processing (25). In addition, there is evidence that microvilli can release membrane vesicles carrying proteins implicated in cell differentiation or cell state, suggesting that microvilli can be a source of membrane microvesicles (28, 29). This phenomenon has also been observed in primary and motile cilia/flagella which function as cellular antennae (30). Although the internal cilium structure is mainly composed of a microtubule-based core architecture, unlike the cross-linked F-actin bundles in microvilli, an emerging body of evidence has demonstrated that cilia can produce microvesicles from their surface (30–34); thus there is a similarity between microvilli and cilia/flagella. Intriguingly, cilia formation has striking similarities to IS formation and ciliary microvesicles contain the endosomal sorting complex required for transport (ESCRT) (34).

Despite the potentially important roles of microvilli, these membrane protrusions have only recently been studied in T cells based on the observation that microvilli are the sites of TCR clustering and thus initial TCR signaling (4, 23, 35). Interestingly, Cai et al. imaged both microvillar footprints and TCR using synaptic contact mapping (SCM) and observed strong co-localization (23). Importantly, they found that TCR microclusters localized on microvilli tips moved centripetally toward central supramolecular activation clusters (cSMAC) (23). The centripetal movements of both microvilli and TCR clusters were also confirmed by fluorescence-tagged protein probes specific for microvilli under total internal reflection fluorescence microscopy (TIRFM) (5). Collectively, these studies suggest that previously observed “microclusters” on a planar surface may reflect activation-dependent, apically clustered TCRs on microvilli; however, there are also controversial reports regarding the relationship between microvilli structure and microcluster formation. Previously it was demonstrated that lymphocyte microvillus morphology does not require Wiskott-Aldrich syndrome protein (WASp) (24), whereas a recent report showed that the generation of actin “foci,” which bridge the early and later stages of TCR signaling, are dependent on WASp (36, 37). Although further detailed studies are required, a possible explanation for this discrepancy could be that WASp does not control the external structure of microvilli, but can functionally affect TCR signaling by forming new F-actin at the very proximal region below early TCR microclusters and recruiting downstream signaling molecules such as phospholipase c-γ (36). Since actin is essential for the centripetal movement of TCR microclusters but appears dispensable for microvilli stabilization (23, 38), it is unlikely that WASp is involved in the persistence of existing microclusters. It is also possible that WASp is required for sorting new TCR molecules on microvilli tips, regardless of the resting or activated state of the T cells.

### Trogocytosis

**Figure 2:**
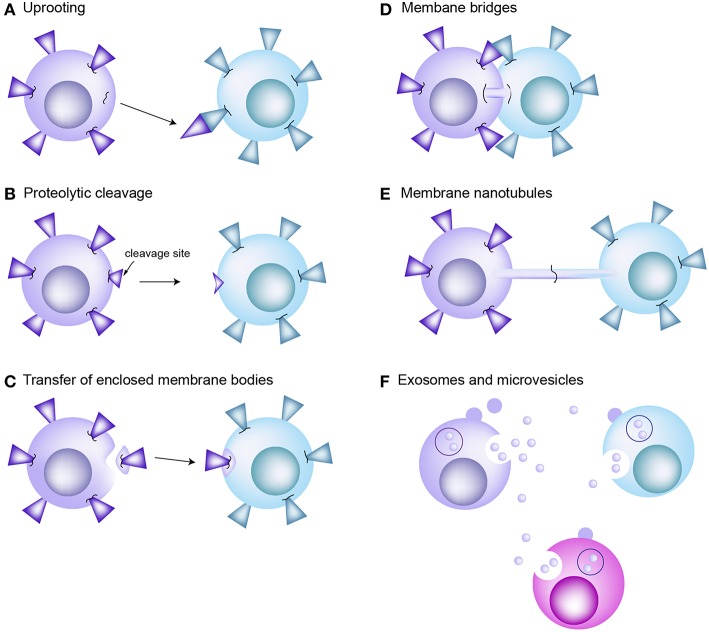
Traditional concepts of intercellular protein transfer between the cells of immune system. **(A)** Uprooting. It has been considered that membrane molecules or patches can be uprooted from plasma membrane and then transferred to the interacting cells as consequence of mechanical force. **(B)** Proteolytic cleavage of membrane molecules. **(C)** Enclosed membrane bodies or vesicles. Transfer of membrane material through vesicle shedding is heavily dependent on interactions between the plasma membrane and underlying cytoskeleton. **(D)** Membrane bridges. Transmission electron microscopy reveals formation of membrane bridges between the CTL and target cell during synapse dissociation. However, there is no evidence that this membrane bridge promotes the exchange of proteins between cells. **(E)** Membrane nanotubes. Tunneling nanotube (TNT) is actin enriched filopodia-like protrusions that may facilitate the exchange of intracellular materials through the actin-dependent mechanism. **(F)** Exosomes and microvesicles carrying transmembrane proteins or RNAs are released from the donor cells. They are participated in long-distance communication. EVs are fused with the plasma membrane of or endocytosed into the recipient cells.

Trogocytosis is a mechanism that rapidly allows the transfer of specific and intact proteins from one cell to another (8, 11). One of the most well-known examples is the transfer of p-MHC complexes from APCs to T cells (10, 23, 39). APCs also acquire TCRs from the T cells (10). Indeed, bidirectional trogocytosis may be commonly occurring during cognate immune cell interactions (10). However, the mechanism of trogocytosis has not been clearly verified; the only proposed mechanism is that cell membrane fragments can be “torn off” by the tensile force generated when cells try to break apart. The high avidity of receptor interactions has been suggested to influence the tearing of membrane patches (40). Figure 2 reveals the traditional concepts of intercellular protein transfer between immune cells including T cells and APCs. However, none of those proposed mechanisms suggested role of microvilli in mediating information exchanges between T cells and APCs.

Recent results from our laboratory have suggested that T cell microvilli are the most easily separable structures during trogocytosis (5). Because the microvilli are enriched with many adhesion molecules in addition to the TCRs and TCR complexes on their tips, microvilli can form multiple bridges with APCs owing to the numerous microvilli on T cell surface (18, 41). Activation of TCR signaling at the microvilli tips can activate adjacent adhesion molecules such as LFA-1, which can meet the conditions that separate microvilli due to the increased receptor affinity and avidity (42). However, a more striking point is that microvilli are selectively enriched with the membrane sorting complexes such as arrestin domain-containing protein 1 (Arrrdc1), TSG101, and Vps4, which are well-known to mediate microvesicle budding at the cell surface and are also known to be involved in the IS (3, 43, 44). This strongly suggests that the generation of microvilli particles is not a passive process but is rather actively mediated by enzyme complexes. Although how TCR signaling is coupled with the fragmentation of microvilli during the cognate interaction of T cells with APCs is currently unknown, the combined action of TCR signaling and adhesion receptor signaling may play critical roles to activate the sorting complex and form small vesicle-like microvilli particles after dissociation from the T cell body. In accordance with this, interestingly, microvilli particles are not significantly generated if T cells are only activated by soluble antibodies against CD3 and CD28. This suggests that TMP generation requires the prior adhesion of T cells on the surface of APCs. This is, indeed, predictable because T cell activation absolutely requires surface antigen peptide presented on an MHC. This fact also suggests that TMPs play a role in the communication with cognate APCs, but not for long-distance communication, which is generally proposed in the research areas of exosomes or microvesicles. However, no production of TMPs without TCR activation suggests that activation of membrane budding complexes needs TCR signaling.

Trogocytosis is not a process limited only to T cells and APCs. Membrane protein exchanges have also been described between many immune cells, including NK cells, B cells, macrophages, and target cells (8, 11, 45). Moreover, it is believed that trogocytosis is a constitutive process in most APCs (46). Therefore, it will be interesting to address whether transfer of proteins in other cell types is also associated with the membrane budding complex. From this point of view, it would be interesting to investigate whether recent discovery of membrane vesicles such as neutrophil trails (46) and migrasomes (47, 48) involve a membrane sorting complex, because these two types of vesicles also need membrane contact via adhesion molecules or the matrix to be generated. In this sense, the term “trogocytosis” is somehow misleading because trogocytosis is based on the concept that membrane patches are “torn off” by the tension generated when cells attempt to move away from each other when engaging in high-avidity protein–protein interactions (11).

### EVs: Exosomes and Microvesicles

Mammalian cells secrete diverse types of membrane vesicles called EVs into the extracellular environment (20, 22, 49, 50). EVs were firstly detected from cultured cells, but have been isolated from more diverse body fluids including blood, milk, urine, semen, saliva, amniotic fluid, ascites fluid, cerebrospinal fluid, broncho-alveolar lavage, and bile (22). However, because there is no clear nomenclature for EVs, these small membrane vesicles can be classified based on their subcellular origins as exosomes and microvesicles (12, 22). Exosomes are stored in the endocytic compartments and released as small vesicles by fusion of late endosomes or multi-vesicular endosomes (MVEs) with the plasma membrane, which then triggers the release of small membrane vesicles. The size of the exosomes is equivalent to that of the intraluminal vesicles of the MVEs from which they originate (40–100 nm in diameter). In contrast, microvesicles are the EVs that bud directly from the plasma membrane and have various other names, such as microparticles, shedding vesicles, microvesicles, and arrestin domain-containing protein 1-mediated microvesicles (ARMMs) (12). Microvesicles are heterogeneous in size and are known to be bigger than exosomes (up to ~1,000 nm in diameter); however, small vesicles (~100 nm) may also bud from the cell surface (51).

There are a number of evidences that indicate that T cells also release exosomes and microvesicles during the formation of ISs (12). However, many previous reports regarding EVs in T cells may have been biased in collecting or naming the EVs. For instance, researchers mostly used an anti-CD3 antibody-coated surface to activate T cells, and then the vesicles from the supernatants were regarded as the exosomes (52). Blanchard et al. demonstrated that TCR activation of human T cells induces the production of exosomes bearing the TCR/CD3/ζ complex. According to our recent work, Rab11^+^ intraluminal vesicles were free of TCR/CD3/ζ complexes. In contrast, particles derived from the microvilli were highly enriched with the TCR complex and other TCR complex proteins. If T cells produce TCR^+^ exosomes from the intracellular compartment by activation, they must produce TCR^+^ exosomes by soluble activators such as soluble anti-CD3 antibody or PMA/ionophore. However, Blanchard et al. and our group demonstrated that T cell stimulation by soluble antibodies or pharmacological agents do not induce the release of EVs, suggesting that exosomes are not likely released mainly from activated T cells. Interestingly, the proteins that Blanchard et al. identified in the exosomes were similar to those discovered in the TMPs.

Another example is the “TCR-enriched microvesicles” reported by Choudhuri et al. (3). They reported that TCR-enriched microvesicles are released from the cSMAC of ISs by membrane budding processes, suggesting that cSMAC is the “secretory domain” of the TCR-enriched microvesicles. However, their claim is somewhat intertwined with that found by Jung et al. (4). The authors demonstrated that microvilli provide a structural platform for TCR clustering and for the recruitment of microclusters upon TCR engagement (4, 35). Indeed, the positioning of preformed TCR clusters in microvilli suggests that the sizes of TCR microclusters are constrained to the physical areas of microvilli tips (35). Moreover, Cai et al. demonstrated that TCR microclusters on microvilli tips migrate due to centripetal actin flow (23). Consistent with the observations by Cai et al. we found that TCR clusters move together with the microvilli signals during IS formation on the lipid bilayer containing p-MHC and ICAM-1 (5). Furthermore, we observed that microvilli signals are accumulated at the cSMAC during IS maturation, confirming that the accumulation of TCR clusters into cSMACs is related to the centripetal movement of microvilli (5). TCR clusters were released from the T cell body when microvilli were detached by the process of trogocytosis. Interestingly, although Choudhuri et al. demonstrated that TCR-enriched microvesicles are released from the cSMAC, we identified that TCR-enriched T cell microvilli particles are highly separated during T cell kinapses, suggesting that the generation of TCR-enriched vesicles are not restricted to the cSMAC, but can be separated from microvilli during T cell activation. Taken together, the currently available data, but not all, strongly demonstrate that both TCR-enriched microvesicles (3) and exosomes (52) might be from the same origin: T cell microvilli.

There is an intriguing report regarding the purification of exosomes from T cells. In contrast to the method described by Blanchard et al. they purified exosomes from cultured cell supernatants under conditions without stimulation (53), presumably assuming that exosomes are naturally secreted from T cells. Interestingly, the authors reported that MVEs were polarized toward the interface between T cells and APCs in an antigen-dependent manner, demonstrating that exosome transit from T cells may occur at the IS (53). The authors claimed that trogocytosis, the transfer mechanism of certain membrane proteins, might be caused by the directional release of exosomes in the IS zone (53). Unfortunately, since the study only characterized the microRNAs in the exosomes, it is unclear whether the exosomes contained TCRs or proteins from TCR complexes that are highly enriched in the TMPs (5, 53). In addition, the authors did not characterize the size of the exosomes deposited on the surface of the recipient B cells, suggesting that the polarized release of exosomes at the IS does not explain the mechanism of trogocytosis (8, 12, 53, 54).

### Membrane Tunneling Nanotubes

Nanotubular highways or tunneling nanotubes (TNT) were first described in neuronal cells, but were seen in a variety of cell types (14, 15, 17, 18, 41, 55–58). TNTs have also been described in immune contexts, including NK cells, macrophages, DCs, monocytes, and Epstein-Barr virus (EBV)-transformed B cells (14, 15, 17). For instance, when monocytes and DCs are stimulated to flux calcium, the signal can be transferred within seconds to other cells hundreds of microns away (59), suggesting a structural connection via TNTs. On the other hand, others suggest that TNTs may be generated upon disassembly of the IS as cells are dissociated (14, 15). If this is the case, it is necessary to reconsider the process of nanotube formation because a previous report suggested that nanotubes are newly formed by membrane protrusions in an active process driven by the actin cytoskeleton (41). However, if nanotubes originate from microvilli, they may not be generated *de novo*; rather, they could be intermediate forms elongated by the “pulling force” of two synaptic cells moving apart. Moreover, evidence for the exchange of proteins via nanotubes between immune cells remains unclear.

We now understand that T cell microvilli produce particles called TMPs. Thus, careful observation of the structural changes of TNTs over a time course may provide an answer whether TNTs are an intermediate structure of microvilli elongation during IS formation or otherwise have their own structure distinct from elongated microvilli. Indeed, many long-distance connections observed between each T cell and surrounding APCs are similar to those observed by the recent works by Kim et al. (5). Interestingly, we found that multiple bridges were more evident during immunological kinapses behind the moving T cells interacting with the antigen-bearing APCs or lipid bilayers (5). Therefore, further studies are required to clarify the relationship between membrane nanotubes and microvillus-originated multiple membrane bridges.

### Immunological Synapses and Kinapses

**Figure 3:**
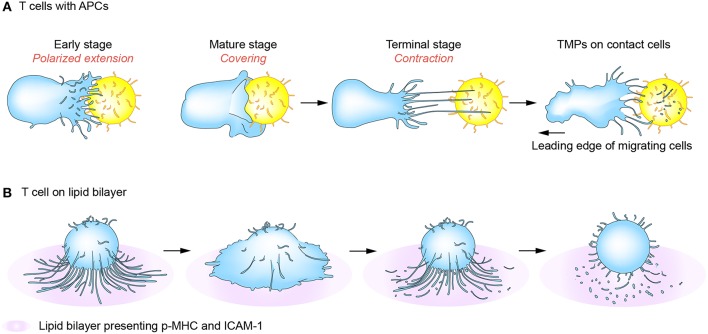
T-cell microvilli particle (TMP) production during immunological synapses (IS) or kinapses. At the early stage of immune synapse formation, numerous TCR-enriched microvilli on T cells are polarized onto the surface of cognate antigen-bearing APCs **(A)** or peptide-MHC-presenting lipid bilayer **(B)** (early stage). When T cells are fully activated, the microvilli mostly disappear but some remain on the outermost edge of mature T cells (mature stage). Tight contact and long microvilli that extend to APCs promote contact-dependent transfer of large microvilli particles. During kinaptic phase, T cells leave abundant T cell microvilli particles on the surface of APCs. Surface anchored microvilli particles are further fragmented to be smallerorigin particles by plasma membrane budding complex such as Arrdc1, TSG101, and Vps4.

T cells search the surface of APCs in a highly dynamic mode and interact with APCs for a variable period from a few minutes to several hours. During this time, T cells alternatively receive “go” and “stop” signals that induce both kinapses and synapses, and T cells recognizing ligands can interconvert these structures periodically (60–62). Interestingly, a recent report showed that most major T cell subtypes spend more time in the kinapse phase, suggesting that durable priming interactions do not necessarily require stable synapses if there is sufficient communication between T cells and DCs (63). In line with this, T cell and DC synapses are known to be composed of several submicronic contact spots and lack large-scale concentric prototypical synapses, suggesting that the concentric structure should not be considered as a “mature synapse.” In this viewpoint, mutual exchange of message-containing conveyors between two cells can be more efficient to sustain the activation status. Interestingly, we observed that T cells in the synaptic phase released relatively few TMPs, but produced more TMPs in kinapses (5). Electron microscopic analysis revealed that regular- and small-exosome-sized TMPs (20–40 nm in diameter) are present in the inner region of the T cell under the transition period between synapse and kinapse. These small and regular TMPs may be the TCR-enriched microvesicles or CD63-positive exosomes which accumulated at the cSMAC as reported previously (3, 53). However, the studies in our laboratory revealed that these regular and small TMPs are surrounded by the irregular and large TMPs (20–500 nm in diameter) which are more evident behind T cells in kinapses (5). The large TMPs are later converted to small-exosome-sized TMPs by the action of membrane budding complexes, such as Arrdc1, TSG101, and Vps4. This strongly suggests that cSMAC is not a secretory domain, but that microvilli tips accumulated at the cSMAC or behind the T cells in kinapses may provide the microdomains for EVs. The generation of TMPs is adhesion-dependent as they are only significantly generated on an adhesive condition mediated by adhesion molecules such as LFA-1/ICAM-1. Collectively, TMP generation requires both TCR-mediated signaling and physical adhesion to the surface of antigen-bearing APCs. Figure 3 reveals a mechanism of TMP generation during IS or immunological kinapses [modified from the paper by Kim et al. (5)]. However, since IS also serves as focal point for TCR internalization, which together acquire MHC class I and II and some costimulatory molecules from APCs (19, 34), it is important to consider how both internalization and secretion (or trogocytosis) occur in the same place. Furthermore, it would be interesting to examine whether TMPs can also be transferred to APCs via the same mechanism as they are internalized in T cells.

### Generation of Microvilli Particles From APCs or Other Immune Cells

As mentioned above, TMPs generation requires the activation of both the TCR and LFA-1 (5). Although DCs possess numerous microvilli on their surface, little is known whether microvilli in DCs can be disconnected and transferred to the surface of T cells. Nevertheless, as trogocytosis of peptide-MHC complexes from DCs to T cells is the best-known mechanism of molecular transfer, it is plausible that microvilli in DCs can be disconnected for transferring messages to T cells. It has been reported that microvilli on DCs exhibit a high density of antigen-presenting and costimulatory molecules (64, 65), implying that DCs also sense and present messages via microvilli. The several submicronic contact spots between T cells and DCs (64, 66) further suggest that microvilli are important for both cells. B cell microvilli are highly enriched with MHC class II and ICAM-1 molecules and possess distinct inducible membrane domains that can control direct cell-cell interactions via the grouping and three-dimensional presentation of cell-surface receptors (67–69). Similarly, NK cells also contain numerous microvilli on their surface (15, 17) and, interestingly, it has been shown that NK cells form submicron scale junctions called nanotubes, in which proteins such as DAP10, the signaling adaptor that associates with the activating receptor NKG2D, and MHC class I chain-related protein A (MICA), a cognate ligand for NKG2D, accumulate (17). These evidences suggest the possibility that NK cell nanotubes are the intermediate structures of elongated microvilli before cells move apart. Thus, it will be interesting to determine whether NK cells also produce microvilli particles in response to the non-specific recognition of target cells. Interestingly, it has been shown that nanotubes can facilitate the lysis of remote target cells (17); however, a previous report demonstrated that the 2B4 molecule (CD244), a co-receptor for human NK cell activation, is clustered at cell-cell contact interfaces between NK cells and target cells and remains bound to the target cell even after NK cell detachment (70). Thus, it is plausible that the 2B4 molecule is clustered at the microvilli tips on the surface of NK cells.

### TMPs Can Be Categorized as a New Class of EVs

In some aspects, TMPs are clearly different from exosomes or microvesicles. First, unlike these two vesicle types, which are released through secretion or membrane shedding, TMPs are necessarily released via a contact- and adhesion-dependent manner. Second, TMPs act only at the surface of physically interacting cells, so it is conceptually different from exosomes or microvesicles, which act over a distance. Therefore, TMP generation is a distinctive and novel system that is more relevant to trogocytosis than to vesicle release by activated T cells. However, it is inappropriate to attribute the formation of TMPs to trogocytosis only because it is also facilitated by the action of membrane budding machinery (12, 28, 44, 71). From this viewpoint, the mechanism of TMP generation is more similar to that of recently identified membrane vesicles, such as neutrophil trails (72) and migrasomes (47), since the generation of these two vesicles also requires membrane contact by adhesion molecules or the matrix (47, 72). We therefore propose that, unlike exosomes and microvesicles which participate in long-distance intercellular communication, TMPs, neutrophil trails, and migrasomes could be defined as a new class of EVs.

### Conclusion and Perspectives

**Figure 4:**
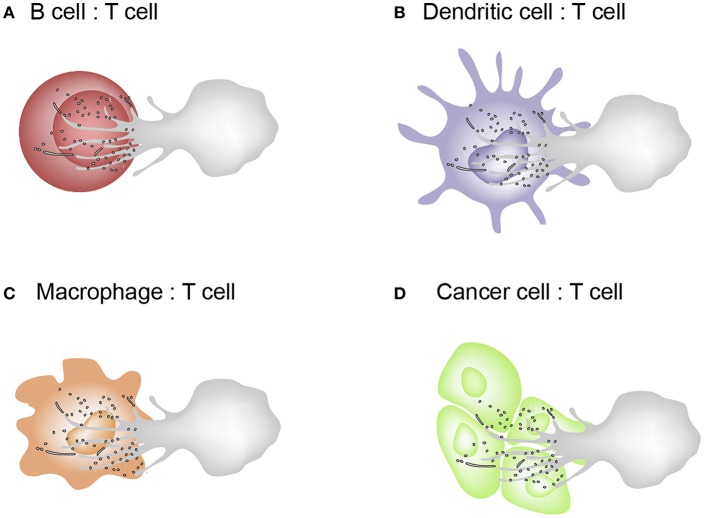
Schematic model describing the exchange of TMPs between T cells and the interacting cells. **(A–C)** T cells interacts with B cells, dendritic cells, and macrophages which are capable of MHC class II–dependent antigen presentation. Cognate interactions between T and interacting cells lead to the formation of the IS where bidirectional activation signals are exchanged. **(D)** Cytotoxic effector T cells recognizes tumor cells or virus-infected cells via the T cell receptor (TCR) and kill them by delivering a cocktail of cytotoxic substances (perforin/granzyme).

T cell microvilli act not only to survey antigens on APCs or target cells but also to send messages to them. The discovery of TMPs suggests the reconsideration of many T cell areas. First, in the past, trogocytosis was considered to have the cell membrane patch torn and transferred when the cells move apart after cell–cell interaction. However, microvilli are where trogocytosis easily occurs and the particles are subsequently released. Second, microvilli contain a large amount of membrane budding complexes, indicating that the membranes are not simply torn, but rather actively fragmented. This is the same mechanism as the budding of viruses. Thus, microvilli tips can be thought of as a place to provide a platform for producing TMPs. Third, these particles can act selectively on cognate antigen-bearing APCs. Because TMPs are only generated upon activation of the TCR and adhesion signals, it can be speculated that TMPs can only regulate the antigen-containing, physically interacting cells *in vivo*. However, TMPs themselves do not have selectivity because, unlike T cell-dependent activation, APCs can be activated through various pathways. The non-selectivity of TMPs implies their potential use in medical applications. For example, as TMPs are able to increase the function of DCs, it can be useful to boost T cell-mediated immune function. TMPs can be the safest immune adjuvant to activate DCs if TMPs are obtained directly from the patient's T cells. Figure 4 represents the schematic model describing the exchange of TMPs between T cells and the interacting cells. This knowledge gives us a better understanding of how TMPs act and the consequences of this mode of communication. In future, TMPs will be promising tools for immunotherapy in immune disorders and in other scenarios if we can engineer the molecules and compositions of TMPs and understand their detailed action mechanisms against target cells or tissues.

## Author Contributions

H-RK wrote and created the figures. C-DJ wrote and finalized the review.

### Conflict of Interest Statement

The authors declare that the research was conducted in the absence of any commercial or financial relationships that could be construed as a potential conflict of interest.
